# Brief Analysis on the Degradation of Sugar-Based Copolyesters

**DOI:** 10.3390/polym15224372

**Published:** 2023-11-10

**Authors:** Dezhi Qu, Ziheng Yang, Jinyu Zhang, Shuyu Wang, Yao Lu

**Affiliations:** 1 Guangxi Key Laboratory of Green Processing of Sugar Resources, College of Biological and Chemical Engineering, Guangxi University of Science and Technology, Liuzhou 545006, China; 2 College of Biological and Chemical Engineering, Guangxi University of Science and Technology, Liuzhou 545006, China; yzh1743165104@163.com (Z.Y.); zhangjinyu2233@163.com (J.Z.); wangshuyu26@163.com (S.W.); 3 Wuxi HIT New Material Research Institute Co., Ltd., Wuxi 214000, China

**Keywords:** isosorbide, monomers, biodegradable polymers

## Abstract

Isosorbide can be used as a third monomer in the synthesis of aliphatic polyesters, and its V-shaped bridging ring structure can effectively improve the rigidity of the copolyester molecular chain. In this work, a series of degradable polyester materials were prepared by modifying polybutylene succinate and using isosorbide as the third monomer. The degradation tests in this paper were implemented through the hydrolysis of copolyesters in distilled water, degradation in natural water and degradation tests in simulated natural environments. The results showed that PBS and its copolyesters can degrade under natural conditions, and the introduction of isosorbide can accelerate the degradation of copolyesters, which could effectively reduce pollutants in nature.

## 1. Introduction

According to the definition of biodegradable polymers by the International Union of Pure and Applied Chemistry, polymers are susceptible to degradation due to biological activity, and their weight decreases with the occurrence of degradation [1,2,3]. Other organizations have supplemented the definition of biodegradable polymers, which can generate carbon dioxide, water and organic matter after degradation [4,5,6].

Polybutylene succinate (PBS) is a biodegradable polyester with good biocompatibility. It is prepared by melt polycondensation of succinic acid and butanediol. PBS is currently mainly used in environmental protection applications, such as films, compostable bags, non-woven fabrics and textiles, catering products and foam [7,8].

PBS can undergo hydrolysis under the catalysis of lipase, esterase and keratinase. PBS is hydrolyzed into butanediol (B), succinic acid (S) as well as water-soluble oligomers mainly composed of SB, SBS, BSB and SBSB [9]. Bai [10] used keratinase to degrade PBS, and found that the weight loss of PBS exceeds 80% when the enzyme degrades for 24 h. Hayase [11] isolated *Bacillus pumilus* 1-A, which could show a degradation rate of 90% within 14 days at 30 °C. Maeda [12] confirmed that *Aspergillus oryzae* RIB40 exhibited significant degradation of PBS within 5 days at 30 °C. Kim MN [13] used sludge to degrade PBS with a degradation rate of 3% in 30 days and 8% in 50 days. Also, HS Kim [14] found that the composting degradation rate of PBS is 12% after 80 days. In this paper, we mainly study the degradation performance of PBS under different conditions, and investigate the impact of the introduction of isosorbide on the degradation of copolyesters [15,16,17,18]. There are currently three main evaluation methods for the degradation performance of degradable polymers [19]. The first takes place in the laboratory, and evaluates the degradation performance of polymers through enzymes and cell methods. The advantage of this method is that it can be evaluated with bacteria and microorganisms in an artificial environment under specified conditions, and the degradation results can be obtained in a relatively short amount of time. The second method uses manual simulation, which mainly uses artificial means to simulate potential problems in the environment, such as water, soil, compost and substances in landfill waste. The advantage of this method is that it can simulate the degradation mode of polymers after being discarded in a natural environment in the laboratory through manual means. The last method uses soil, in which the polymer is buried directly in the soil and the degradation of polymer in the actual environment is continuously tracked to achieve the aims of this research. The advantage of this method is that the polymer degrades in a natural environment, and the results are reliable.

In this paper, the degradation of copolyesters under three conditions, distilled water, natural water and a natural environment simulation, was evaluated and analyzed by analyzing the obtained results, such as their weight loss, viscosity average molecular weight, carboxyl end group and crystallization melting enthalpy. In addition, the influence of isosorbide (in terms of PBS degradation) was also evaluated.

## 2. Materials and Methods

### 2.1. Materials and Synthesis

#### 2.1.1. Materials

Potassium hydroxide (KOH), ethanol (EtOH), phenol and tetrachloroethane were purchased from Nanjing Chemical Reagent Co., Ltd. (Nanjing, China). 1,4-butanediol (BDO) was supplied by Jiangsu Yongfeng Chemical Reagent Co., Ltd. (Danyang, China). Succinic acid (SA) was purchased from Shandong Feiyang Chemical Co., Ltd. (Xintai, China). Isosorbide was supplied by Hubei Yuanchenggongchuang Co., Ltd. (Wuhan, China). Ethylene glycol stibium (EGSb) was purchased from Shanghai Qizhi Chemical Co., Ltd. (Shanghai, China). All regents were used directly without any purification.

#### 2.1.2. Synthesis

PBS and its copolyesters were synthesized with the condensation polymerization process. SA and BDO with the molar ratio of 1:1.2 were added into a 1 L polyester synthesis reactor, and BDO was replaced with isosorbide in a certain proportion during the synthesis of copolyesters. EGSb was used as a catalyst with the amount of 0.05 wt% based on the total weight of SA, BDO and isosorbide. The esterification reaction was carried out at 190–220 °C under a nitrogen atmosphere. The reaction system was slowly reduced to 30 Pa within 1 h and the reaction temperature was increased to 245 °C for polycondensation. After a continuous reaction for 6 h at 245 °C in a vacuum of 30 Pa, PBS was obtained. In the abbreviation (PBISx), x refers to the molar percentage (mol%) of isosorbide relative to the total diols.

### 2.2. Methods

#### 2.2.1. Hydrolysis Degradation Test

Weigh the polyester sample and place it in distilled water for degradation in a 20 °C water bath shaker. After a certain interval time, take out the sample and dry the surface moisture, and then dry it in a vacuum oven at 50 °C for 4 h. There are at least three samples for each time of testing.

(1) $$\begin{array}{c} weight\;loss=\frac{m_{0}\;-\;m_{1}}{m_{0}}\times 100\% \end{array}$$

where *m*_0_ is the mass of sample before degradation, and *m*_1_ is the mass of sample after several days of degradation and after being dried in the vacuum oven.

#### 2.2.2. Degradation Test in Natural Water

Weigh the polyester sample and place it in natural water (Wuxi, China) for degradation in a water bath shaker. After a certain interval time, take out the sample and dry the surface moisture, and then dry it in a vacuum oven at 50 °C for 4 h. The weight loss is calculated with Equation (1). There are at least three samples for each time of testing.

#### 2.2.3. Simulated Natural Environment Test

The degradation of polymers under natural conditions mainly includes photodegradation, which is caused via exposure to ultraviolet radiation in sunlight as well as hydrolysis (after being washed with rain). In order to simulate the degradation reaction of copolyesters after being discarded under natural conditions, an accelerated weathering tester was used to simulate the degradation of copolyesters under natural conditions.

Place the polyester sample in an accelerated weathering tester (QUV, Q-Lab, Westlake, OH, USA) and use the ASTM-G154-07 [20] method for testing, which comprises the following three steps. First, the irradiation intensity of ultraviolet light is 1.55 W/m^2^, and it is irradiated for 8 h to simulate daily light with the temperature of 60 °C. Second, spray the sample with distilled water for 15 min to simulate rain flushing. Finally, set the temperature to 50 °C to simulate an environment where water vapor condenses at night. Perform these three steps for a total of 12 h for cyclic testing. After a certain interval time, take out the sample and dry the surface moisture, and then dry it in a vacuum oven at 50 °C for 4 h.

#### 2.2.4. Viscosity Average Molecular Weights of Copolyesters

Dissolve 0.125 g of polyester in 25 mL mixed solvent of phenol and tetrachloroethane (1:1 mass ratio), and test using a 0.8 mm Ubbelohde viscometer in a water bath at 25 °C. Calculate the intrinsic viscosity of polyester according to the following equations:

(2) $$\begin{array}{c} \eta_{SP}=\frac{t_{1}\;-\;t_{0}}{t_{0}} \end{array}$$

(3) $$\begin{array}{c} \left[\eta \right]=\frac{\sqrt{1\;+\;1.4\eta_{sp}}\;-\;1}{0.7c} \end{array}$$

(4) $$\begin{array}{c} \left[\eta \right]=KM_{\eta }^{\alpha } \end{array}$$

where *η_sp_* is specific viscosity, *t*_1_ is time of solution flow (s), *t*_0_ is time of solvent flow (s), [η] is intrinsic viscosity (dL/g), *c* is concentration of polyester solution (g/100 mL), *M_η_* is viscosity average molecular weight (g/mol) and K and α are 1.71 × 10^−4^ and 0.788 [21].

#### 2.2.5. Determination of Carboxyl End Group Content

Dissolve the polyester in 50 mL mixed solvent of phenol and tetrachloroethane (1:1 mass ratio), and titrate the polyester solution using an ethanol solution of potassium hydroxide (KOH-EtOH), and calculate the carboxyl end group content of copolyester using the equation.

(5) $$\begin{array}{c} D=\frac{(V\;-\;V_{O})\;\times \;c\;\times \;10^{3}}{m} \end{array}$$

where D is the amount of carboxyl end group in polyester (mol/t), *V*_0_ is volume of KOH-EtOH consumed by blank solution of phenol and tetrachloroethane (mL), *V* is volume of KOH-EtOH consumed by polyester solution (mL), *c* is the concentrate of KOH-EtOH (mol/L), *m* is the weight of copolyester (g).

#### 2.2.6. Crystallization Melting Enthalpy Test

Take about 5 mg of sample after degradation and use the differential scanning calorimeter (200 F3, NETZSCH, Selb, Germany) for testing. Under a nitrogen atmosphere, the temperature rises from room temperature at a rate of 10 K/min to 150 °C. And the crystallization melting enthalpy was calculated by integrating the melting peak area.

#### 2.2.7. Mechanical Strength Testing

The tensile strength and elongation at the breakpoint of polyester were tested on a universal mechanical machine (E44, MTS, Eden Prairie, MN, USA) with a tensile rate of 10 mm/min, according to the ASTM 638-14 [22], by using 5 samples for testing.

#### 2.2.8. Morphology Testing

The microscopic morphology of copolyester after degradation was observed using scanning electron microscope (SUPRA55, Zeiss, Jena, Germany). Moreover, the samples were treated with gold before testing.

The optical morphology of copolyester after degradation was carried out with a microscope (BX51, Olympus, Tokyo, Japan) with incident and reflected light sources.

## 3. Results

### 3.1. Hydrolytic Behavior in Water

#### 3.1.1. Weight Loss Rates of Copolyesters

**Figure 1:**
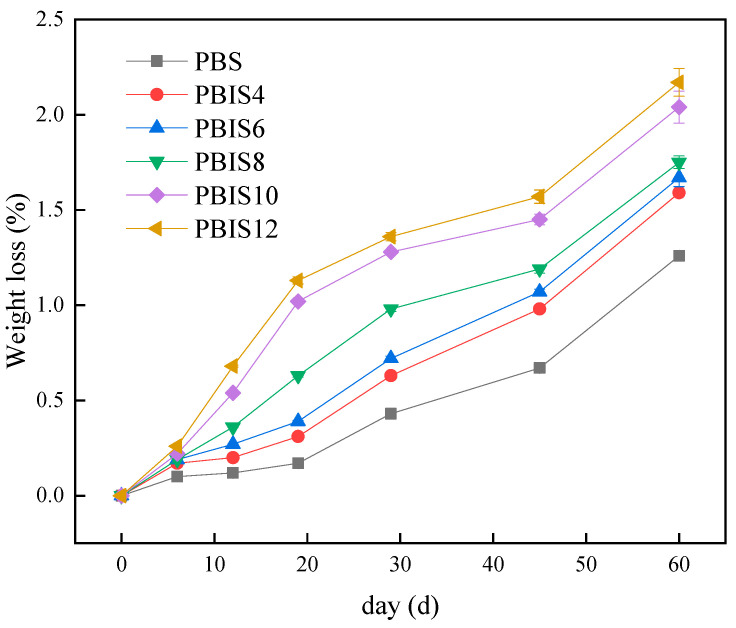
Weight loss of copolyesters in distilled water.

**Figure 2:**
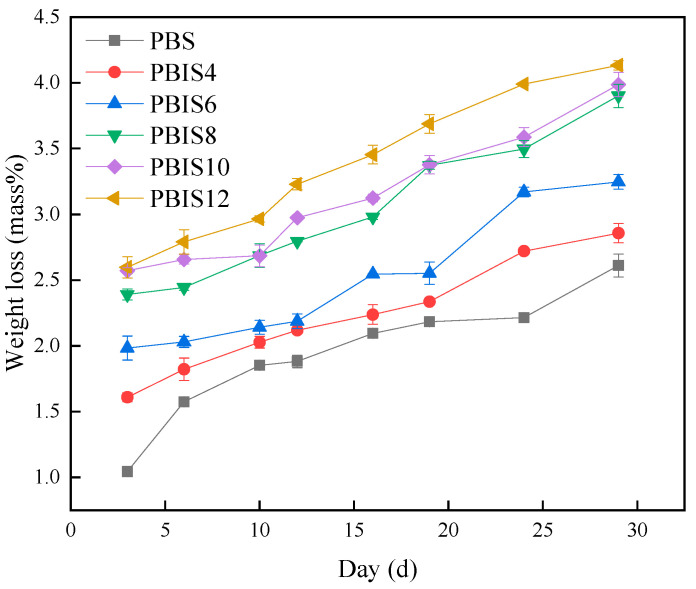
Weight loss of PBIS in natural water.

The weight loss rate curves of PBS and copolyesters in water at 20 °C are shown in Figure 1. It can be seen from Figure 1 that the weight loss rates of PBS and copolyesters are higher with the increase in time. The weight loss rate of PBS at 6 days, 19 days, 29 days and 60 days were 0.10%, 0.17%, 0.43% and 1.26% in distilled water, respectively. Furthermore, the weight loss rate shows an increasing trend with the addition of isosorbide content; the weight loss rate of PBIS12 was 2.17% at 60 days, which indicates that the introduction of isosorbide will improve the hydrolysis ability of copolyesters; the greater the isosorbide content, the more obvious the acceleration effect on the hydrolysis reaction. The curves in Figure 1 have shown that the weight loss rates of copolyesters does not increase too much at the initial stage of degradation. The weight loss rate increases greatly when the degradation time exceeds 19 days, and the overall weight loss rate shows a trend of first smoothing and then accelerating. This is due to the high molecular weight of PBS and its copolyesters at the initial stage of degradation, and the wetting process of water on the polymer surface also takes a certain time. However, it can be seen from Figure 1 that the platform period of PBIS10 and PBIS12 is not very obvious when the isosorbide content exceeds 10 mol%. Due to the influence of isosorbide on the surface polarity of copolyesters, PBIS10 and PBIS12 have an affinity for water which is easy to infiltrate on the surface, and the platform period is not obvious. According to the Deng et al. [23], the hydrolysis of polymer starts from the surface, and the greater the affinity of polymer to water, the faster the hydrolysis. Therefore, the introduction of isosorbide improves the affinity between copolyesters and water; thus, water can fully soak on the surface of copolyesters and improves their hydrolysis rates. In addition, the introduction of isosorbide will reduce the crystallinity of copolyesters. According to the Tserki et al. [24], the lower the crystallinity of polymer, the faster the degradation rate. Therefore, the influence of isosorbide on the crystallinity of copolyesters will also accelerate the degradation of copolyesters. In Figure 2, it can be seen that the weight loss rates of copolyesters show an increasing trend with the increase in degradation times, and there are obvious weight losses within 3 days. The obvious weight losses are caused by the rapid decomposition of small molecules in copolyesters with bacteria and microorganisms, and the degradation rates are stable when they are completely decomposed. The weight loss rate of PBS was 2.61%, while that of PBIS12 was 4.13% in 29 days. The stable degradation rate mainly exists because of the catalysis process performed by the bacteria and enzymes which are produced by the microorganisms in natural water. Compared with the degradation in distilled water, the degradation of copolyesters in natural water is obviously greater than that in distilled water. For example, the weight loss in distilled water is 1.35%, while its weight loss in natural water is 4.15% when PBIS12 was degraded for 29 days. It shows that microorganisms and bacteria in natural water have an obvious catalytic effect on the degradation of copolyesters. The results shows that the content of isosorbide will affect the degradation of copolyesters in natural water. According to Deng et al. [23], it is difficult to attach bacteria and microorganisms to the surface with low polarity. Therefore, increasing the polarity of a copolyester’s surface with isosorbide will improve the adhesion ability of bacteria and microorganisms and the more significant promotion effect on the degradation reaction of a copolyester.

#### 3.1.2. Viscosity Average Molecular Weights of Copolyesters

Table 1 shows the changes in viscosity average molecular weights of copolyesters during different hydrolysis periods. The viscosity average molecular weights of copolyesters gradually decrease with the increases in hydrolysis times. This shows that the molecular weights of copolyesters decrease gradually under this condition, which means that copolyesters can hydrolyze under this condition. Moreover, the increase in isosorbide content will increase the decrease of the molecular weights of copolyesters, which indicates that isosorbide can accelerate the hydrolysis of copolyesters. The viscosity average molecular weights of copolyesters degraded in natural water is significantly lower than those degraded in distilled water. For example, the viscosity average molecular weight of PBIS8 in distilled water for 19 days is 139,800 g/mol, while in natural water it is 113,000 g/mol, which illustrates that the degradation degree of copolyesters in the natural water is greater than that in distilled water. As the content of isosorbide increases, the molecular weight reductions of copolyesters increase. This is mainly due to the introduction of isosorbide, which improves the hydrophilicity of copolyesters, and can quickly soak water and effectively accelerate the action of water on copolyesters.

#### 3.1.3. Carboxyl End-Group Content of Copolyesters

Table 2 shows the changes in carboxyl end-group contents of copolyesters during different hydrolysis periods. It can be seen from Table 2 that copolyesters have a low carboxyl end group-content before degradation. This due to the excess in the glycol monomer during the synthesis reaction; thus, the end group of its molecular chain is hydroxyl. However, a small amount of the carboxyl end group still exists in the polyester synthesis, such as polyethylene terephthalate (PET), which will contain a small amount of the carboxyl-terminated group. However, due to the poor stability of the carboxyl group of succinic acid, it will produce a decarboxylation reaction at the temperature of polycondensation, which results in a very low content of carboxyl end groups in copolyesters. At the same time, the acid will promote the hydrolysis of polyesters. Therefore, the carboxyl end group in the molecular chain can further promote the hydrolysis of copolyesters and accelerate the hydrolysis of copolyesters when they undergo degradation reactions. Based on the data in Table 2, it can be concluded that the amount of carboxyl end groups increases significantly as the content of isosorbide increases. Meanwhile, the degradation rate in natural water is significantly faster than in distilled water. According to the Harder et al. [4], the degradation of polyester in a natural environment is mainly achieved through the enzymes’ actions on the ester bonds of polyester, which are secreted with bacteria and microorganisms. Therefore, the degradation of copolyesters in natural water is still essentially a hydrolysis reaction. Enzymes serve as catalysts for the hydrolysis of copolyesters, which can effectively catalyze the hydrolysis reaction of copolyesters.

#### 3.1.4. Crystallization Melting Enthalpy of Polyester

Table 3 lists the crystallization melting enthalpy of PBS and copolyesters measured with DSC under different hydrolysis periods. From Table 3, it can be seen that the crystallization melting enthalpy shows an upward trend with the increase in degradation time, which indicates an increase in the proportion of crystalline parts per gram in the copolyester sample. According to the result, the hydrolysis reaction of PBS and copolyesters mainly occurs in the amorphous region, which leads to an increase in the proportion of the crystalline region. According to the weight loss (Figure 1 and Figure 2), the increase in the crystallization melting enthalpy is slightly higher than the weight loss rate. As the test temperature is higher than the glass transition temperature of the copolyester samples, the amorphous region of the sample can continue to generate a small amount of crystallization under the testing environment, and the small molecular segments generated with main chain degradation will also participate in crystallization, which results in a slightly higher increase in the melting enthalpy than the weight loss rate. From the data in Table 3, it can be seen that the melting enthalpy changes of different copolyesters before and after degradation are roughly the same. Therefore, it can be considered that the recrystallization phenomenon during the degradation process is not closely related to the content of isosorbide. In other words, the recrystallization that occurs during the degradation of copolyesters is composed of molecular fragments which are composed of succinic acid and butanediol.

#### 3.1.5. Surface Morphology of Polyester

**Figure 3:**
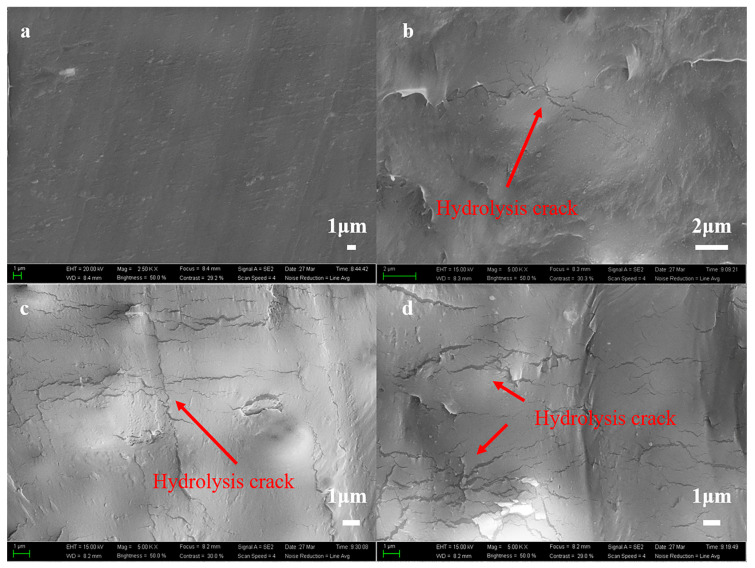
SEM of PBIS10 surface morphology after degradation; (**a**) 0 days; (**b**) 12 days; (**c**) 45 days; (**d**) 60 days.

The surface morphology of copolyesters after hydrolysis was analyzed with scanning electron microscopy. Figure 3 shows the SEM of the surface morphology of PBIS10 after degradation in distilled water for 0 days, 12 days, 45 days and 60 days. From Figure 3a, it can be seen that the surface of the copolyester is smooth and flat before hydrolysis. After 12 days of degradation in distilled water, the cracks began to appear on the surface of the copolyester. At 45 days of degradation, it can be seen that the surface of the copolyester is filled with a large number of cracks, indicating that a large amount of degradation had already began to occur on the surface. When the degradation reaches 60 days, the cracks on the surface of the copolyester continue to expand.

**Figure 4:**
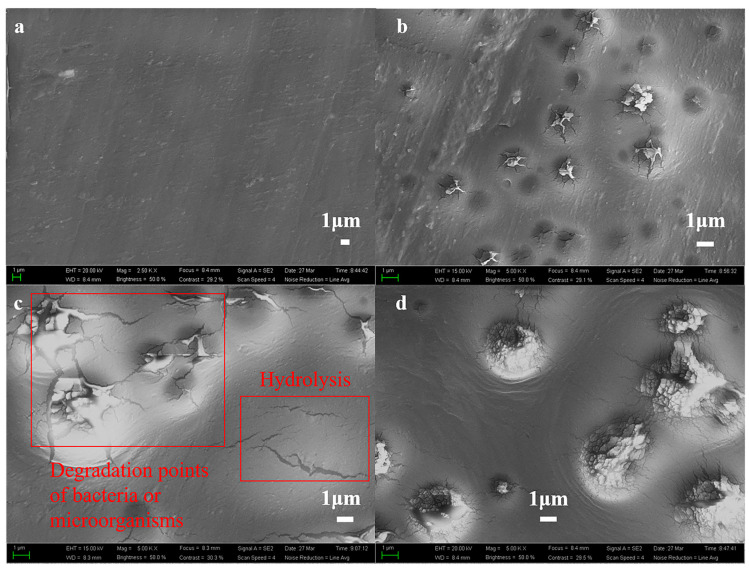
SEM of PBIS10 surface morphology after degradation in natural water; (**a**) 0 days; (**b**) 19 days; (**c**) 24 days; (**d**) 29 days.

Figure 4 shows the SEM of PBIS10 at 19 days, 24 days and 29 days of degradation in natural water. A large number of point-like protrusions were generated on the surface when PBIS10 was degraded in natural water for 12 days, and there were many cracks on the protrusion surface. When the degradation reaches 29 days, the protruding cracks increase in diameter to reach 2 μm, and the polyester in these cracks has shown fragmented morphology. These presented degradation points indicate that the degradation of copolyesters in natural water bodies begins at the sites where bacteria and microorganisms attach. Bacteria and microorganisms attach to the surface and enzymes secreted at the attachment points degrade copolyesters to produce small molecule substances for their survival. From Figure 4c, it can be seen that the surface of copolyesters has degradation point-like protrusions generated by bacteria and microorganisms, as well as cracks generated with the self-hydrolysis of copolyesters, which indicate that copolyesters undergo both bacterial and microbial catalytic hydrolysis and self-hydrolysis in natural water.

#### 3.1.6. Mechanical Properties of Copolyesters

**Figure 5:**
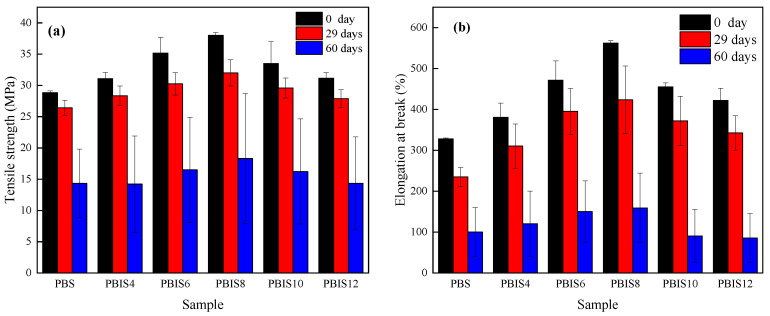
Mechanical properties of copolyesters degraded in distilled water. (**a**) Tensile strength; (**b**) elongation at breakpoint.

Figure 5 shows the changes in tensile strength and elongation at the breakpoint of polyesters at different degradation times in distilled water. In Figure 5, it can be found that the tensile strength and elongation at the breakpoint of copolyesters gradually decrease with the increase in degradation time; however, the decrease in elongation at the breakpoint is more obvious. The tensile strength and elongation at the breakpoint of copolyesters showed a slight decrease after degradation for 29 days. When degraded for 60 days, the tensile strength of copolyesters decreases to about 50% of its original value, while the elongation at the breakpoint significantly decreases to only about 25% of its original vale. The error range of both tensile strength and elongation at the breakpoint is very wide after degradation for 60 days. According to the analysis of crystallization melting enthalpy, the degradation of copolyesters occurs in the amorphous region. The amorphous region, which serves as a bridge between crystal regions, gradually degrades and disappears during the degradation process. When copolyesters are under external forces, there is not enough of continuous phases between crystal regions to disperse the external forces, which easily cause fractures at weak points, causing a decrease in the mechanical strength of copolyesters. The location of the degradation of copolyesters is uncontrollable, and the location and degree of degradation between each test sample are not the same, which can lead to significant fluctuations in the testing error range of copolyesters after degradation. From the results, it can be inferred that the addition of isosorbide can improve the mechanical properties of the copolyester film; however, an excess quantity of isosorbide can also accelerate the decline in the mechanical properties of copolyester film, especially the elongation at their breakpoints.

**Figure 6:**
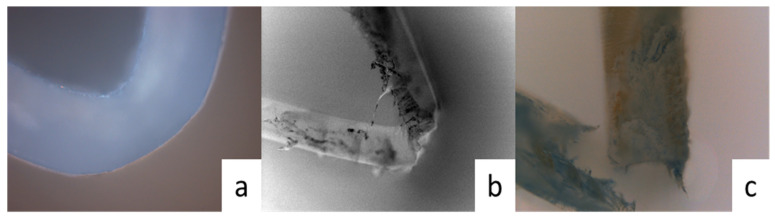
Picture of PBIS8 bending after degradation in natural water at (**a**) 0 days; (**b**) 19 days; (**c**) 29 days.

Due to the fast degradation rate of copolyesters in natural water and the large number of degradation points, their mechanical properties significantly decrease in a short period of time and have a large error range, which make them impossible to measure. During the test of the degradation of polyesters in natural water, we found that their flexibility can be observed with our own eyes. Figure 6 shows the bending image of a copolyester in natural water. From the figure, it can be seen that the copolyester film has good flexibility before degradation, and it exhibits good toughness at the bending point. After degradation for 19 days, the copolyester film fractured at the bending point; however, a portion of copolyester film remained in a continuous phase and did not completely fracture. When the degradation time reached 29 days, the copolyester film completely fractured at the bending point, which indicated that the degradation has reduced the toughness of that copolyester. According to the result, the degradation in the amorphous region is the main culprit.

### 3.2. Degradation Performance of Copolyesters in Simulated Natural Environments

**Figure 7:**
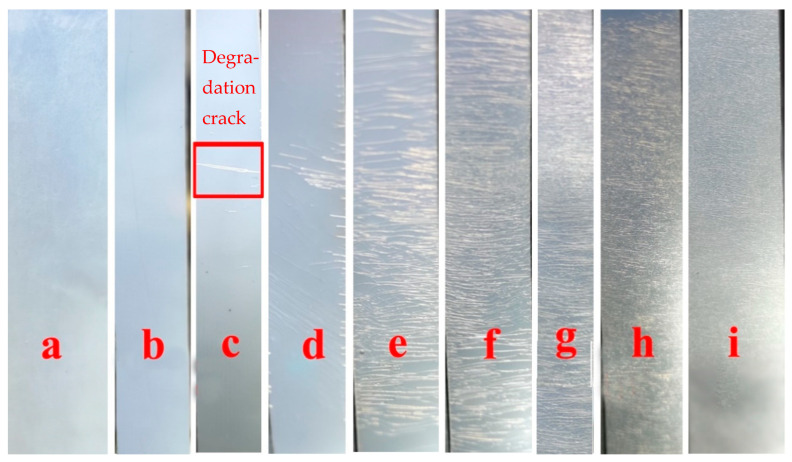
The pictures of PBIS8 tested in accelerated weathering texter for (**a**) 0 h; (**b**) 24 h; (**c**) 48 h; (**d**) 72 h; (**e**) 96 h; (**f**) 120 h; (**g**) 144 h; (**h**) 168 h; (**i**) 192 h.

Figure 7 show pictures of PBIS8 tested in an accelerated weathering tester. It can be seen from the figure that the copolyester did not visually change within the first 24 h. When the testing time reached 48 h, cracks began to appear inside the film sample, and the cracks gradually increased with the increase in testing time. The testing area was already covered with cracks when the testing time reached 192 h.

**Figure 8:**
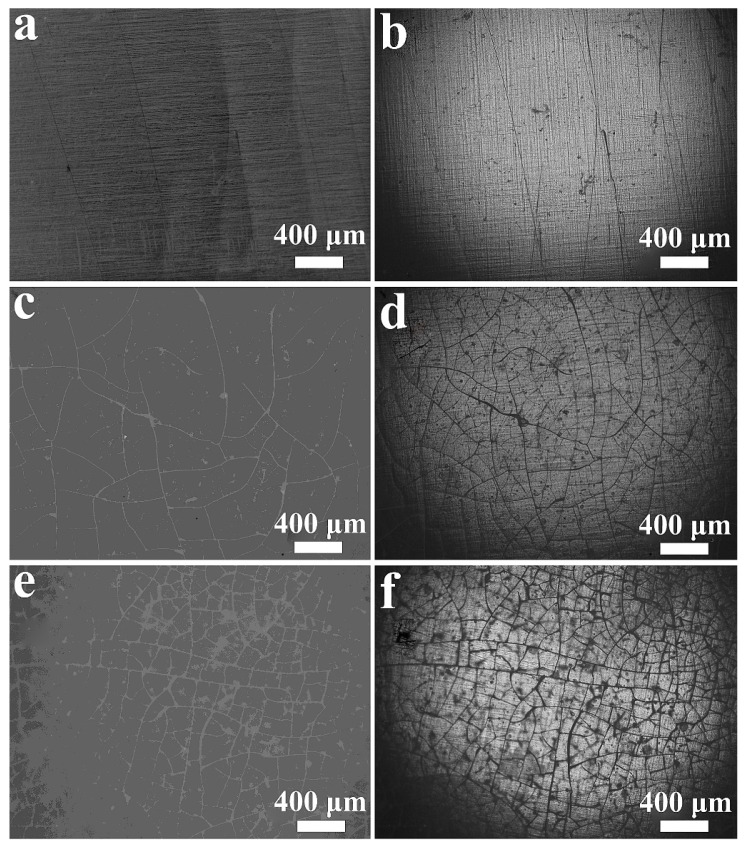
Pictures taken with optical microscope of PBIS8 tested in accelerated weathering texter at (**a**) 24 h, reflection; (**b**) 24 h, transmission; (**c**) 72 h, reflection; (**d**) 72 h, transmission; (**e**) 144 h, emission; (**f**) 144 h, transmission.

Further observations of the microstructure of cracks in PBIS8 in the accelerated weathering tester were carried out using an optical microscope, as shown in Figure 8. The surface morphology of the copolyester film was observed using a reflection light of a microscope, and the interior of the copolyester film was observed using a transmission light. The results indicate that only slight cracks were observed on the surface of the copolyester during 24 h of testing. When the testing time reached 72 h and 144 h, cracks could also be observed inside the copolyester test film samples, indicating that the degradation of the copolyester under this condition is not limited to the surface due to the transmission of ultraviolet light. The hydrolysis of copolyesters under this condition occurs simultaneously through surface erosion and internal erosion, and the results show that it occurs uniformly on the surface and inside of the polymer. This mainly includes two factors. Firstly, when ultraviolet light completely penetrates the polyester film, it will provide energy to the chemical bonds, which could cause molecular chain breakage and cracking. Secondly, it is the diffusion of water on the surface and cracks which triggers hydrolysis reactions in the amorphous areas of the polymer. The oligomers produced with the internal degradation of the polyester film slowly diffuse to the polymer surface through the cracks and gradually overflow. It can also be seen from Figure 8 that there are significantly more surface cracks than internal cracks, indicating that the rate of surface erosion is much higher than internal erosion because the erosion starts from the polymer surface.

## 4. Conclusions

In this research, the degradation of poly(ethylene-co-isosorbide succinate) was investigated in the following three conditions: distilled water, natural water and simulated natural environments. The results show that the isosorbide will accelerate the hydrolysis rate of copolyesters, with the content of isosorbide increasing with the increase in acceleration. The results from the micrograph indicated that the performance of hydrolysis results is cracks on the surface of the polyester. Moreover, when bacteria and microorganisms attach to the surfaces, degradation begins at the attach points. Finally, the simulation of the degradation of copolyesters indicated that the degradation which combines the action of ultraviolet light and water is faster than the degradation of copolyesters in distilled water and natural water.

## Figures and Tables

**Table 1:** Changes of viscosity average molecular weights of copolyesters.

	Day/d	Viscosity Average Molecular Weight/g·mol^−1^					
		PBS	PBIS4	PBIS6	PBIS8	PBIS10	PBIS12
	0	159,600 (200)	158,300 (700)	160,300 (400)	159,400 (400)	161,200 (800)	157,500 (1000)
Distilled Water	19	145,600 (1000)	141,500 (700)	143,200 (800)	139,800 (2000)	142,500 (1200)	132,400 (1200)
	29	132,600 (2400)	135,300 (1100)	129,500 (1200)	113,500 (1600)	125,800 (1100)	113,500 (700)
	45	123,000 (2700)	115,000 (2100)	105,300 (1800)	108,500 (2900)	112,500 (1600)	94,300 (3000)
	60	102,300 (3500)	103,500 (3700)	97,500 (3400)	96,500 (4000)	92,000 (4600)	89,500 (3600)
Natural Water	19	121,600 (1300)	121,900 (2400)	117,500 (1700)	113,000 (2500)	107,500 (1500)	102,400 (1300)
	29	89,500 (5100)	87,200 (6000)	88,700 (6200)	82,600 (7200)	73,700 (4800)	73,200 (6000)

The values in the parentheses represent the error range of molecular weight.

**Table 2:** Carboxyl end-group content of copolyesters before and after degradation.

	Degradation Time/d	Carboxyl End Group/mol·t^−1^					
		PBS	PBIS4	PBIS6	PBIS8	PBIS10	PBIS12
	0	1.8	2.1	1.7	1.6	1.3	2.1
Distilled Water	29	245.2 (37.6)	269.4 (35.3)	273.8 (25.3)	271.6 (41.0)	288.3 (20.3)	296.5 (26.1)
	60	475.7 (97.8)	504.3 (58.6)	517.5 (58.8)	531.4 (75.5)	540.2 (59.2)	548.7 (58.1)
Natural Water	19	186.6 (21.1)	207.4 (35.9)	218.6 (14.1)	249.0 (28.0)	275.4 (14.6)	290.2 (40.5)
	29	510.8 (124.8)	533.3 (92.8)	536.1 (119.8)	586.6 (86.6)	628.4 (62.0)	653.9 (142.8)

The values in the parentheses represent the error range of carboxyl end group.

**Table 3:** Changes of crystallization melting enthalpy of copolyesters before and after hydrolysis.

	Degradation Time/Days	Crystallization Melting Enthalpy/J·g^−1^					
		PBS	PBIS4	PBIS6	PBIS8	PBIS10	PBIS12
	0	67.22	64.52	62.46	54.15	52.11	50.49
Distilled Water	12	67.72	64.80	62.89	54.57	52.85	50.91
	29	68.37	65.45	63.28	54.97	53.17	51.43
	45	68.83	66.01	63.94	55.41	53.78	51.96
	60	69.66	66.52	64.76	56.01	54.67	52.54
Natural Water	6	68.24	65.26	63.64	54.86	53.26	52.03
	12	69.02	65.91	64.84	55.80	64.32	52.73
	19	69.97	66.49	65.27	56.62	55.14	53.47
	29	70.42	67.87	66.49	57.86	56.15	54.59

## Data Availability

The data presented in this study are available on request from the corresponding author.
